# Protein-Losing Enteropathy in Systemic Lupus Erythematosus: 12 Years Experience from a Chinese Academic Center

**DOI:** 10.1371/journal.pone.0114684

**Published:** 2014-12-09

**Authors:** Zhen Chen, Meng-Tao Li, Dong Xu, Hong Yang, Jing Li, Jiu-Liang Zhao, Heng-Hui Zhang, Shao-Mei Han, Tao Xu, Xiao-Feng Zeng

**Affiliations:** 1 Department of Rheumatology, Peking Union Medical College Hospital, Chinese Academy of Medical Science & Peking Union Medical College, Beijing, China, and Key Laboratory of Rheumatology and Clinical Immunology, Ministry of Education, Beijing, China; 2 Department of Gastroenterology, Peking Union Medical College Hospital, Chinese Academy of Medical Science & Peking Union Medical College, Beijing, China; 3 Peking University People’s Hospital, Peking University Hepatology Institute, Beijing Key Laboratory of Hepatitis C and Immunotherapy for Liver Diseases, Beijing, China; 4 Department of Epidemiology and Statistics, Chinese Academy of Medical Science & Peking Union Medical College, Beijing, China; Nippon Medical School Graduate School of Medicine, Japan

## Abstract

**Objective:**

Protein-losing enteropathy (PLE) is a complication in some systemic lupus erythematosus (SLE) patients that is often misdiagnosed. With this study, we provide insight into clinical characteristics, laboratory characteristics, diagnostic tests, risk factors, treatment, and prognosis of the disease.

**Methods:**

A retrospective, case-control study was performed in 44 patients with SLE-related PLE (PLE group) and 88 patients with active SLE (control group) admitted to our care from January 2000−January 2012. Risk factors for SLE-related PLE were examined, and we analyzed the accuracy of single and combined laboratory characteristics in discriminating SLE-related PLE from active SLE. Serum albumin and C3 levels were measured as outcome during and after treatment with corticosteroids and immunosuppressive agents.

**Results:**

The PLE group had lower mean serum albumin and 24-hour urine protein levels, higher mean total plasma cholesterol levels, and greater frequencies of anti-SSA and SSB seropositivity compared with the control group. Anti-SSA seropositivity, hypoalbuminemia, and hypercholesterolemia were independent risk factors for SLE-related PLE. The simultaneous presence of serum albumin (<22 g/l) and 24-hour urine protein (<0.8 g/24 h) had high specificity, positive predictive value, negative predictive value, and positive likelihood ratio, a low negative likelihood ratio and no significant reduction in sensitivity. High dosage of glucocorticosteroid combined with cyclophosphomide were mostly prescribed for SLE-related PLE.

**Conclusion:**

SLE-related PLE should be considered when an SLE patient presents with generalized edema, anti-SSA antibody seropositivity, hypercholesterolemia, severe hypoalbuminemia, and low 24-hour urine protein levels. Aggressive treatment for lupus might improve prognosis.

## Introduction

Protein-losing enteropathy (PLE) is a condition characterized by profound edema and severe hypoalbuminemia that is secondary to excessive serum protein loss from the gastrointestinal (GI) tract [1]. Hypoalbuminemia is an important clinical symptom as it can lead to edema, fluid balance disorders, and heart failure. In addition, patients with hypoalbuminemia are vulnerable to infection. In systemic lupus erythematosus (SLE), hypoalbuminemia is usually ascribed to nephrotic syndrome, disease exacerbation, or liver disease [2]. Most PLE-related GI manifestations are not as common as other organ involvement such as lupus nephritis, and PLE is clinically indistinguishable from nephrotic syndrome [3]. Therefore, PLE can be easily missed by internists, rheumatologists, gastroenterologists, and nephrologists [2].

Most previous reports on PLE describe isolated cases or small case series of patients [4]–[28]. Because little information exists on SLE-related PLE, we undertook a case-control study to: (1) describe the clinical and laboratory characteristics of this complication in patients with SLE; (2) explore risk factors for PLE in SLE patients and evaluate the discriminative ability of laboratory characteristics; and (3) report our experience on the treatment of SLE-related PLE with a combination corticosteroid and immunosuppressive cyclophosphamide therapy.

## Methods

### Patients

All patients were admitted to the Peking Union Medical College Hospital (PUMCH) between January 2000 and January 2012. A total of 44 patients were enrolled in the PLE group. Patients diagnosed with SLE according to the American College of Rheumatology (ACR) 1997 revised criteria were included in the study [29]. PLE diagnoses were based on clinical symptoms and laboratory test results often encountered in patients with PLE, irrespective of Technetium 99 m-labelled (99 m Tc) human serum albumin (HSA) scintigraphy results. For this study, a clinical diagnosis of PLE included hypoalbuminemia that could not be fully explained by urinary protein loss, and reduced protein synthesis in which malnutrition or severe liver disease had been excluded. Patients that had evidence of protein leakage from the GI tract as detected by 99 m Tc-HSA scintigraphy were considered to have definite PLE, whereas patients in which this examination was not performed were considered to have probable PLE. The control patient group comprised 88 active SLE patients that were randomly and consecutively selected by SPSS software among the 4648 contemporaneous SLE patients (without PLE) at the PUMCH. Patients with concurrent Sjögren’s syndrome were identified according to American European Consensus Group (AECG) classification criteria [30]. Written informed consent was obtained from all patients prior to admission to our hospital, and the study was approved by the PUMCH ethics committee.

### Clinical and laboratory data

Demographic (gender, age, disease duration) and clinical data for all patients were collected according to Systemic Lupus International Collaborating Clinics (SLICC) 2012 criteria [31]. Laboratory data included: serum albumin and C3 and C4 complement levels; plasma calcium, total cholesterol, and triglyceride levels; and 24-hour urine protein levels. Autoantibody profiles (anti-nuclear antibody [ANA], anti-double-stranded DNA antibody [anti-dsDNA], anti-ENA antibodies [including anti-SSA, anti-SSB, anti-Sm, and anti-RNP antibody]), and Systemic Lupus Erythematosus Disease Activity Index 2000 (SLEDAI-2K) [32] were obtained from all patients upon admission to hospital. ANA was detected by indirect immunofluorescence (IIF) with HEp-2 cell substrates. Anti-dsDNA was detected by IIF using flagellate protista substrates and enzyme-linked immunosorbent assay (ELISA). Anti-ENA was detected by immunodiffusion assay.

### Treatment and assessment of clinical response

All patients in the PLE group were treated with corticosteroids. This treatment was administered intravenously. Initial intravenous steroid dosage was 1 mg/kg/d prednisone or the equivalent dosage of another corticosteroid. For methylprednisolone (MP) pulse therapy, 1000 mg was administered for three days and then changed to intravenous prednisone 1 mg/kg/d. Immunosuppressive therapy was administered as required. Immunosuppressive agents included cyclophosphamide (CTX), mycophenolate mofetil (MMF), and methotrexate (MTX).

Complete response (CR) to therapy for the PLE group was defined as total resolution of edema and GI symptoms, together with return of serum albumin levels to ≥35 g/dl. Partial response (PR) was defined as partial improvement of edema and GI symptoms with improved serum albumin levels (≥30 g/dl). Non-response (NR) was defined as the failure of serum albumin levels to increase to 30 g/dl or more [13].

### Statistical analyses

SPSS software v.17 (Chicago, USA) was used for all statistical analyses. To analyze differences between groups, Student’s t test or Wilcoxon rank test were used for numerical data. The Chi square test or Fisher’s exact test was used for qualitative data. Variables with significant *P* values between groups in the univariate analysis were computed to a multivariate logistic regression model to predict independent risk factors for SLE-related PLE. The discriminative ability of laboratory tests was assessed using either the standard threshold, i.e., the threshold reported in the literature or judged to be clinically meaningful, or the best threshold, i.e., the threshold obtained through the receiver operating characteristic (ROC) curve analysis that produced the most appropriate tradeoff between sensitivity and specificity [33]. The ability of single and combined in series laboratory characteristics to discriminate PLE from active SLE was evaluated by ROC curve analysis. Sensitivity, specificity, positive predictive values (PPV), negative predictive values (NPV), positive likelihood ratios (PLR), and negative likelihood ratios (NLR) were determined. Two-tailed *P* values <0.05 were considered statistically significant.

## Results

### Clinical characteristics

A total of 44 patients with SLE-related PLE were included in the PLE group. Twenty-six patients had probable PLE and 18 patients had definite PLE. Patients in this group were predominantly women (84.1%). The mean age was 35.1±2.13 years (range, 16–71 years). These data were comparable with the control group (Table 1). However, patients with PLE had greater frequencies of serositis and hypocomplementemia compared with patients in the control group. Upon admission to the hospital, patients in the PLE group had lower frequencies of acute cutaneous lupus, synovitis, and anti-dsDNA seropositivity compared with the control group. At diagnosis, renal and neurological symptoms, as well as the proportion of anti-Sm–positive patients were similar between groups. The mean SLEDAI-2K score of all patients within the PLE group was significantly lower than in the control group (8.0±0.75 vs. 10.4±0.59, *P* = 0.015). Eight (18.2%) patients in the PLE group and eight (9.09%) patients in the control group had concomitant Sjögren’s syndrome. The frequency of Sjögren’s syndrome was not different between the two groups (*P* = 0.131).

**Table 1:** Demographic characteristics and clinical data according to SLICC criteria in the PLE and control groups*.

Group	PLE (N = 44)	Control (N = 88)	*P*
**Female sex n, (%)**	37 (84.1)	70 (79.5)	0.530
**Age, years (mean ± SE)**	35.1±2.13	34.0±1.48	0.671
**SLE Disease duration, months (mean ± SE)**	42.1±9.56	50.5±8.51	0.543
**PLE disease duration, months (mean ± SE)**	11.9±3.20	–	–
**SLICC criteria at admission, n (%)**			
** Acute cutaneous lupus**	6 (13.6)	32 (36.4)	0.007
** Chronic cutaneous lupus**	1 (2.27)	1 (1.14)	1.000
** Oral ulcers**	1 (2.27)	13 (14.8)	0.058
** Non-scarring alopecia**	5 (11.4)	13 (14.8)	0.591
** Synovitis**	6 (13.6)	31 (35.2)	0.009
** Serositis**	34 (77.3)	18 (20.5)	<0.001
** Renal**	25 (56.8)	58 (65.9)	0.308
** Neurologic**	5 (11.4)	14 (15.9)	0.483
** Hemolytic anemia**	1 (2.27)	1 (1.14)	1.000
** Leukopenia**	6 (13.6)	21 (23.9)	0.170
** Thrombocytopenia**	7 (15.9)	24 (27.3)	0.147
** ANA**	44 (100)	88 (100)	1.000
** Anti-dsDNA**	10 (22.7)	43 (48.9)	0.004
** Anti-Sm**	2 (4.55)	11 (12.5)	0.256
** Antiphospholipid**	2 (4.55)	9 (10.2)	0.436
** Low complement**	39 (88.6)	63 (71.6)	0.028
** Direct Coombs’ test**	3 (6.82)	5 (5.68)	1.000
**SLEDAI-2K score**	8.0±0.75	10.4±0.59	0.015
**Concurrent Sjögren’s syndrome**	8(18.2)	8(9.09)	0.131

*Except when otherwise indicated, values are expressed as counted data (%). SLEDAI-2K: Systemic Lupus Erythematosus Disease Activity Index 2000; SLICC: Systemic Lupus International Collaborating Clinics.

Thirty-nine (88.6%) patients in the PLE group had different degrees of peripheral pitting edema. Seven (15.9%) patients had nausea and vomiting; 15.9% and 50.0% of patients had abdominal pain and diarrhea, respectively. Thirty-nine (88.6%) patients had ascites, 33 (75.0%) had pleural effusion, and 22 (50.0%) had pericardial effusion. Upon determination of organ involvement, 12 (27.3%) patients in the PLE group had single GI involvement, and 32 patients (72.7%) had concomitant disease activity in other organs. Twenty-five PLE patients (56.8%) had concomitant lupus nephritis (LN). Of these, seven patients underwent renal biopsy. Histopathological analyses revealed two patients with Class II LN, one patient with Class IV LN, and four patients with Class V LN. Of the 25 concomitant LN patients, seven patients had 24-hour urine protein secretion >0.5 g, including one patient with minor pathologic changes in the renal biopsy, and six patients who demonstrated protein leakage from the GI tract by 99 m Tc-HSA scintigraphy, indicating that the serum albumin decline observed in these patients was mainly caused by loss from the GI tract.

Five (11.4%) patients’ conditions in the PLE group were complicated with neuropsychiatric syndromes of SLE (NPSLE), four had severe hematologic abnormalities, two had lung involvement, and three had angiogenesis with embolization. The distribution of SLEDAI-2K scores between patients in the PLE group was as follows: six patients scored >12, thirty-four patients scored between 4–12, and four patients scored between 1–3 (S1 Table in S1 File).

### Laboratory tests

Mean serum albumin, C3 and C4, plasma calcium, and 24-hour urine protein levels were markedly lower in the PLE group compared with the control group. Mean platelet count and total plasma cholesterol levels were markedly higher in the PLE group compared with the control group. Twenty-nine (65.9%) patients with PLE were anti-SSA-seropositive and this percentage was higher than in the control group. Twenty five percent of patients in the PLE group were anti-SSB–seropositive, which was also higher than in the control group. The percentage of anti-RNP-positive patients and mean plasma triglyceride levels were comparable between groups (Table 2).

**Table 2:** Laboratory findings in the PLE and control groups*.

Group	PLE (N = 44)	Control (N = 88)	*P*
**Platelet count (×10^9^/l)**	243.9±19.6	180.3±10.5	0.002
**Albumin, (g/l)**	16.8±0.79	30.5±0.91	<0.001
**Plasma calcium (mmol/l)**	1.85±0.02	2.15±0.02	<0.001
**Total cholesterol (mmol/l)**	7.12±0.46	5.97±0.30	0.039
**Triglyceride (mmol/l)**	3.58±0.58	2.43±0.18	0.067
**Serum C3, g/l**	0.47±0.03	0.63±0.03	0.001
**Serum C4, g/l**	0.09±0.008	0.13±0.01	0.01
**24-hour urine protein (g/24** **h)**	0.50±0.11	2.97±0.40	<0.001
**Anti-SSA, n (%)**	29 (65.9)	39 (44.3)	0.019
**Anti-SSB, n (%)**	11 (25.0)	6 (6.82)	0.003
**Anti-RNP, n (%)^#^**	8 (22.2)	19 (23.8)	0.857

*Except when otherwise indicated, data are expressed as mean values ± SE. ^#^ For anti-RNP, data were available for 36 patients in the PLE group, and 80 patients in the control group.

### Probable PLE vs. definite PLE

The differences between definite PLE and probable PLE patients within the PLE group were examined. The demographic characteristics of patients with definite PLE and patients with probable PLE were comparable, as were mean values or frequencies of laboratory test results and the frequency of all SLICC criteria upon admission to the hospital. However, neurological symptoms were more common in patients with definite PLE. In addition, patients with definite PLE had borderline lower serum complement levels, yet higher SLEDAI-2K scores compared to patients with probable PLE (S1−S3 Tables in S1 File).

### Risk factors for SLE patients with PLE

Variables with significant *P* values between groups in single factor analyses were computed using a multivariable logistic regression model to predict the independent risk factors for SLE-related PLE (Table 3). Anti-SSA seropositivity, hypoalbuminemia, and hypercholesterolemia were independent risk factors for SLE-related PLE, while hypoalbuminemia and hypercholesterolemia were negatively associated with SLE-related PLE. Therefore, the odds of an SLE patient with anti-SSA seropositivity developing PLE were 3.718 times higher than an SLE patient who was anti-SSA-negative. If the SLE patient was anti-SSB-positive, the odds of developing PLE were as high as 7.225, but this value was not statistically significant (*P* = 0.08).

**Table 3:** Multivariable logistic regression analysis to predict risk factors for SLE-related PLE.

Variable	*P* value	OR	95% CI
**Anti-SSA antibody**	0.046	3.718	1.025–13.49
**Hypoalbuminemia**	<0.001	0.701	0.593–0.828
**Hypercholesterolemia**	0.005	0.709	0.557–0.904
**Anti-SSB**	0.08	7.225	0.789–66.137
**Plasma calcium**	0.24	0.127	0.004–3.982

CI: 95% confidence interval; OR: odds ratio.

### Diagnostic test evaluation

As with the mean values for the laboratory parameters described above, the frequency of laboratory abnormalities was much greater for patients in the PLE group compared with the control group. Exceptions included comparable frequencies of higher plasma triglyceride levels and lower serum C4 levels (S4 Table in S1 File).

Sensitivity, specificity, and the area under the ROC curves for each laboratory characteristic, assessed using the standard threshold or the best threshold obtained through ROC curve analysis are shown in Table 4.

**Table 4:** Sensitivity and specificity of laboratory parameters analyzed for the ability to discriminate SLE-related PLE from active SLE without PLE*.

	Standardthreshold	Se	Sp	Bestthreshold	Se	Sp	AUC (95% CI)
**Albumin (g/l)**	<35	100	40.9	<22	86.4	80.7	0.90(0.85–0.96)
**Plasma calcium (mmol/l)**	<2.13	95.5	55.7	<1.93	81.8	84.1	0.88(0.83–0.95)
**24 hour urine protein (g/24** **h)**	<0.5	79.5	64.8	<0.8	90.9	56.8	0.73(0.64–0.81)
**Total cholesterol (mmol/l)**	>5.7	65.9	59.1	>6.9	59.1	71.6	0.62(0.51–0.73)
**Triglyceride (mmol/l)**	>1.7	77.3	38.6	>2.07	68.2	54.5	0.63(0.54–0.73)
**Serum C3 (g/l)**	<0.6	81.4	44.0	<0.48	60.5	64.3	0.64(0.54–0.74)
**Serum C4 (g/l)**	<0.12	72.5	43.2	<0.07	40.0	73.8	0.58(0.48–0.69)
**Platelet count (×10^9^/l)**	>300	29.3	92.0	≥246	53.7	79.5	0.65(0.54–0.76)

*Sensitivity and specificity were obtained using a standard threshold (thresholds previously reported in the literature or judged to be clinically meaningful). For the best threshold, the threshold value was obtained through ROC curve analysis that produced the most appropriate tradeoff between sensitivity and specificity. Se: sensitivity; Sp: specificity; AUC: area under the curve; 95% CI: 95% confidence interval.

Plasma total cholesterol and triglyceride levels yielded similar sensitivity and specificity using the standard or the best threshold. Platelet count; serum albumin, C3, and C4; plasma calcium, and 24-hour urine protein levels yielded different sensitivity and specificity depending on whether the standard or best threshold was used. Overall, hypoalbuminemia had the best sensitivity and specificity, followed by decreased plasma calcium levels and 24-hour urine protein levels.

We sought the combination of laboratory variables in series that had the greatest diagnostic accuracy. The laboratory variables included were serum albumin, plasma calcium, and 24-hour urine protein levels. For each combination of variables, sensitivity, specificity, PPV, NPV, PLR, and NLR were calculated (Table 5). The best results were obtained using the simultaneous detection of serum albumin (<22 g/l) and 24-hour urine protein levels (<0.8 g/24 h), which yielded sensitivity 0.818, specificity 0.989, PPV 0.973, NPV 0.916, PLR 74.36, and NLR 0.184.

**Table 5:** Sensitivity and specificity of laboratory parameters combined in series and analyzed for ability to discriminate SLE-related PLE from active SLE without PLE.

	Se	Sp	PPV	NPV	PLR	NLR
**Albumin <22** **g/l and 24-hour** **urine protein <0.8** **g/24** **h**	0.818	0.989	0.973	0.916	74.36	0.184
**Albumin<22** **g/l and Plasma** **calcium <1.93** **mmol/l**	0.773	0.875	0.756	0.885	6.184	0.259
**Plasma calcium <1.93** **mmol/l** **and 24-hour urine protein <0.8** **g/24** **h**	0.773	0.978	0.944	0.896	35.14	0.232

NLR: negative likelihood ratio; NPV: negative predictive value; PLR: positive likelihood ratio; PPV: positive predictive value; Se: sensitivity; Sp: specificity.

### Endoscopy and imaging

Twenty-four patients (54.5%) in the PLE group underwent esophagogastroduodenoscopy (OGD). Of these, 23 (95.8%) had chronic superficial gastritis, generalized mucosal edema, and diffuse non-erosive erythematous GI mucosa. Approximately one-third of the PLE patients (13/44) underwent abdominal contrast-enhanced computed tomography (CT) scans. Circumferential bowel wall thickening due to submucosal edema was documented in 10 (76.9%) of 13 patients. A 99 m Tc-labeled HSA scan documented protein leakage in 18 (100%) of patients (S5 Table in S1 File).

### Treatment and prognosis

All patients in the PLE group received intravenously administered corticosteroid therapy. The initial steroid dosage was 1 mg/kg/d of intravenously administered prednisone or the equivalent dosage of another corticosteroid. Fourteen patients (31.8%) had 1000 mg methylprednisolone (MP) pulse therapy for three days and then changed to intravenous prednisone (1 mg/kg/d). Forty (90.9%) of 44 patients received immunosuppressive therapy. Cyclophosphamide (CTX) was the most commonly administered immunosuppressive medication (37 cases), followed by mycophenolate mofetil (MMF, 1 case), and methotrexate (MTX, 1 case).

Eighteen cases were followed-up for longer than six months. After two months of treatment, eight (44.4%) of these patients had CR, four (22.2%) had PR and six (33.3%) had NR prognoses. Sixteen (88.9%) patients had CR after six months of treatment and the total response rate was 88.9% (S6 Table in S1 File).

**Figure 1:**
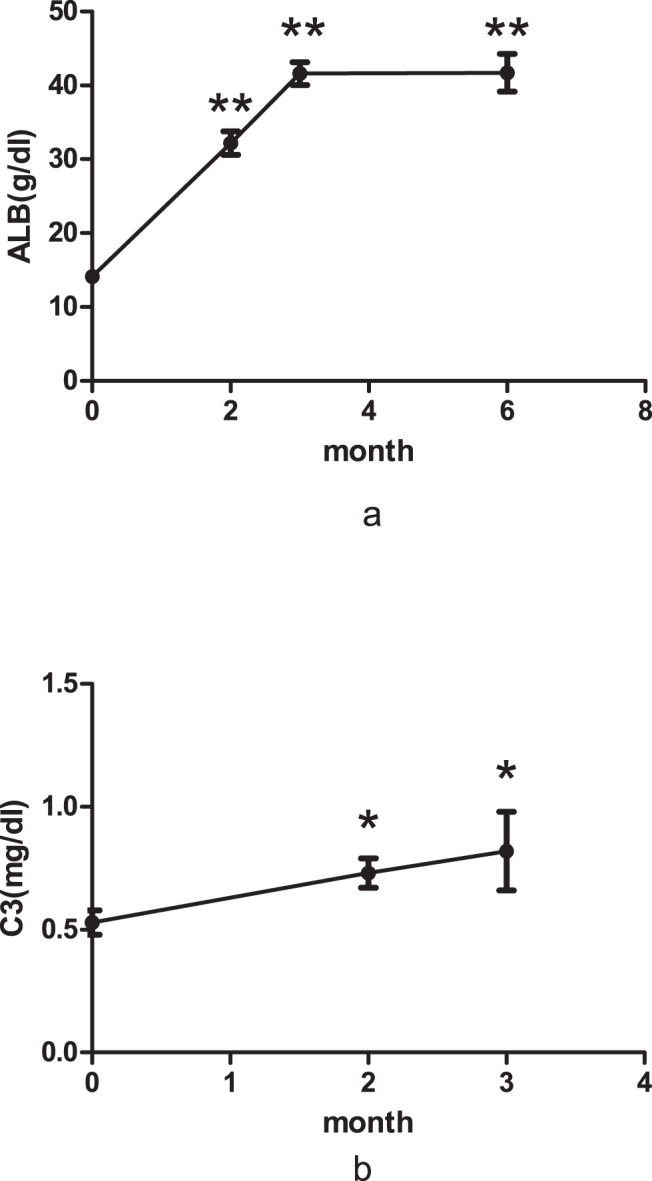
Serum albumin (a) and C3 (b) levels in PLE patients during treatment and follow-up. Data are expressed as mean ± standard error of mean (SE). Asterisks indicate statistically significant differences from the values at baseline; ** = *P*<0.001, * = *P*<0.05.

We documented the changes in serum albumin and C3 levels in the PLE patients during treatment (Fig. 1). A significant elevation in serum albumin was noted in the first two months (*P*<0.001), which was accompanied by a significant elevation in serum C3 levels (*P* = 0.028). Serum albumin levels gradually increased, reaching maximal levels during the third month of treatment (*P*<0.001).

## Discussion

We documented the clinical presentation, diagnoses, treatment, and prognoses of 44 SLE-related PLE patients that were admitted into our care over a 12 year period. To our knowledge, this is the first case-control study of lupus-related PLE reported in the literature.

Although no specific epidemiological data exist for SLE-related PLE, it is estimated that the point prevalence of PLE is 3.2–7.5% of Chinese individuals with SLE in Hong Kong [4], [13]. We previously reported that the prevalence of SLE-related PLE in a smaller case series was 1.9% [12]. In this study, we extended the study period to 12 years and surveyed 4692 SLE patients that attended the lupus clinics at our hospital, thus decreasing the prevalence to 0.94%. This figure may be an underestimation as screening for protein leakage from the GI tract is not routinely performed unless there is a clinical indication for unexplained hypoalbuminemia [13].

Aoki et al. have previously concluded that lupus patients might initially present with PLE symptoms, and Gornisiewicz et al showed that PLE often occurs in patients with clinically severe SLE [25], [26]. In the current study, close to 30% of the PLE patients had single GI involvement as the initial manifestation of SLE. This is a reminder that gastroenterologists should be aware of the possibility of SLE whenever this syndrome is encountered. Over 70% of the PLE patients in our case series had other organ involvement; LN was the most common, followed by hematologic involvement, and NPSLE. Therefore, PLE can occur in SLE patients with multiple system involvement or it can occur alone. Our data are consistent with other the reported cases in which common presenting features of SLE-related PLE are edema, while GI symptoms were infrequent [4], [12], [13].

We also found that SLE-related PLE patients had hypoalbuminemia, hypocalcemia, hypercholesterolemia, and C3 and C4 hypocomplementemia, and 23% of patients had anti-dsDNA seropositivity. The frequency of anti-dsDNA seropositivity reported in previous studies ranges from “negative in most cases” [12] to 52% (25/48) [4], 57% (4/7) [10], and 75% (12/16) [13]. Future studies are required to further elucidate the frequency of anti-dsDNA seropositivity in SLE-related PLE patients.

We note that the positive rate of anti-SSA/SSB was much higher in patients with SLE-related PLE. We confirmed this phenomenon through case-control analysis, and these data are consistent with those of Zheng et al [12]. Interestingly, anti-SSA/SSB seropositivity is associated with columnar epithelium cells of labial and salivary glands [34]. Whether anti-SSA/SSB acts on intestinal columnar epithelium cells and causes damage is unknown, and this possibility requires further examination.

PLE is clinically indistinguishable from other complications such as nephrotic syndrome [3]. Furthermore, PLE and LN can successively or simultaneously appear in a SLE patient. In our study, over two thirds of PLE patients had concomitant organ manifestation of SLE upon admission. This further increases the difficulty of diagnosing PLE. With this in mind, we performed a risk factor analysis and diagnostic test evaluation. In the multivariate logistic regression analysis, anti-SSA seropositivity was an independent risk factor, and it was positively associated with SLE-related PLE.

Most laboratory tests provided high sensitivity, but low specificity. Some showed excellent discriminating properties with use of the best threshold. The laboratory characteristics that had the strongest ability to discriminate SLE-related PLE from active SLE were hypoalbuminemia and hypocalcemia, whose sensitivity and specificity were approximately 85%, and AUC were approximately 0.9. To decrease the misdiagnosis rate, we explored the accuracy of laboratory characteristics that were serially combined. The best results were obtained using the simultaneous presence of serum albumin (<22 g/l) and 24-hour urine protein (<0.8 g/24 h). This combination had higher specificity, PPV, NPV, and PLR, lower NLR, and no significant reduction in sensitivity.

99 m Tc-HSA scintigraphy is regarded as the diagnostic tool of choice for patients with PLE [2], [3], [35], as it has very high sensitivity for diagnosis and localization of PLE [36]–[38]. However, its application is limited because many hospitals are not able to perform the procedure. Furthermore, some cases show negative results during disease duration. Law et al. reported that two SLE patients in their cohort had negative scans, but PLE was later confirmed by fecal alpha-1-antitrypsin clearance (AATC) tests [4]. We compared the typical clinical and laboratory characteristics of PLE in patients with definite and probable PLE. Almost all characteristics were comparable, including anti-SSA seropositivity, albumin, and total cholesterol level. This finding suggests that SLE-related PLE can be diagnosed in the absence of evidence from 99 m Tc-HSA scintigraphy, and a negative scintigraphy result does not negate a PLE diagnosis.

The exact pathogenesis of SLE-related PLE is unclear. The postulated mechanisms include mucosal ulceration, increased mucosal capillary permeability as a result of intravascular activation and conversion of complement, complement- or cytokine-mediated (e. g. tumor necrosis factor-α and interleukin-6) vascular or mucosal damage, and ruptured mucosal lacteals resulting in lymphangiectasia [1]–[3], [23].

Corticosteroids and immunosuppressants were the mainstay treatment for SLE-related PLE patients in our study. Because of low cost efficacy, CTX is one of the most commonly used immunosuppressants for SLE in China. However, there are no studies demonstrating CTX efficacy for the treatment of SLE-related PLE. Most SLE-related PLE patients respond well to combined prednisolone and azathioprine (AZA) [4], [13]. In our study, almost 90% of our patients responded well to corticosteroids and immunosuppressive therapy within six months. Serum albumin and C3 levels significantly increased after two months of treatment, and by the third month, serum albumin had achieved normal levels and remained stable until six months after treatment. However, it remains unclear whether steroid monotherapy or combination therapy with cyclophosphamide is appropriate for SLE-related PLE patients.

Nonetheless, our study should be interpreted along with several limitations. First, all patients in this study came from a single healthcare center, and this raises the possibility of a referral bias. Second, data collection was conducted retrospectively. Third, the number of cases was too small to allow global conclusions to be drawn from the results of some of the statistical tests. Fourth, a relatively short follow-up period is unfavorable for comprehensive evaluation of this treatment. The main strengths of our study lie in the case-control population, and in the number of patients with SLE-related PLE.

In summary, PLE is an uncommon complication of SLE. It can present as an initial manifestation of SLE, or accompany other organ damage that is often observed in SLE patients. Anti-SSA seropositivity, hypoalbuminemia and hypercholesterolemia were independent risk factors for SLE-related PLE patients. When a patient with SLE presents with generalized edema, severe hypoalbuminemia, and low levels of 24-hour urine protein, SLE-related PLE should be considered. Results from 99 m Tc-HSA scintigraphy may be negative even in the presence of typical PLE clinical and laboratory characteristics. Aggressive treatment for lupus had a high response rate.

## Supporting Information

S1 File **This file contains S1–S6 Tables.** **S1 Table.** Frequency of clinical characteristics of patients with definite and probable PLE. **S2 Table.** Demographic characteristics and clinical data according to SLICC criteria in patients with definite and probable PLE. **S3 Table.** Laboratory findings in patients with definite and probable PLE. **S4 Table.** Frequency of laboratory characteristics in the PLE and control groups. **S5 Table.** Endoscopic and imaging findings from patients in the PLE group. **S6 Table.** Treatment and prognosis of PLE patients.
(DOCX)
