# Supplementary material for: Protein-Losing Enteropathy in Systemic Lupus Erythematosus: 12 Years Experience from a Chinese Academic Center
Source: PLoS One. 2014 Dec 9;9(12):e114684. doi: 10.1371/journal.pone.0114684 (PMC4260872; doi:10.1371/journal.pone.0114684)
Supplement: S1 File — This file contains S1–S6 Tables. S1 Table. Frequency of clinical characteristics of patients with definite and probable PLE. S2 Table. Demographic characteristics and clinical data according to SLICC criteria in patients with definite and probable PLE. S3 Table. Laboratory findings in patients with definite and probable PLE. S4 Table. Frequency of laboratory characteristics in the PLE and control groups. S5 Table. Endoscopic and imaging findings from patients in the PLE group. S6 Table. Treatment and prognosis of PLE patients. (DOCX) [file pone.0114684.s001.docx]

Supplemental tables

Supplemental table 1. Frequency of clinical characteristics of patients with definite and probable PLE*

| Symptoms and signs | | PLE group  (N=44) | | Probable PLE  (N=26) | | Definite PLE  (N=18) | *P*^δ^ |  |
| --- | --- | --- | --- | --- | --- | --- | --- | --- |
| Peripheral edema | | 39 (88.6) | | 23 (88.5) | | 16 (88.9) | 1.000 |  |
| Nausea and vomiting | | 7 (15.9) | | 4 (15.4) | | 3 (16.7) | 1.000 |  |
| Abdominal pain | | 7 (15.9) | | 3 (11.5) | | 4 (22.2) | 0.594 |  |
| Diarrhea | | 22 (50.0) | | 15 (57.7) | | 7 (38.9) | 0.220 |  |
| Fever | | 8 (18.2) | | 5 (19.2) | | 3 (16.7) | 1.000 |  |
| Lymphadenectasis | | 15 (34.1) | | 14 (53.8) | | 1 (5.56) | 0.001 |  |
| Ascites | | 39 (88.6) | | 24 (92.3) | | 15 (83.3) | 1.000 |  |
| Pleural effusion | | 33 (75.0) | | 19 (73.1) | | 13 (72.2) | 1.000 |  |
| Pericardial effusion | | 22 (50.0) | | 15 (57.7) | | 7 (38.9) | 0.220 |  |
| Single GI involvement | | 12 (27.3) | | 8 (30.8) | | 4 (22.2) | 0.778 |  |
| Concomitant organ manifestation  of SLE on admission | | 32 (72.7) | | 18 (69.2) | | 14 (77.8)) | 0.778 |  |
| Lupus nephrosis | | 25 (56.8) | | 14 (53.8) | | 11 (61.1) | 0.632 |  |
| II | | 2 | | - | | - | - |  |
| IV | | 1 | | - | | - | - |  |
| V | | 4 | | - | | - | - |  |
| Hematological disturbance | | 10 (22.7) | | 6 (23.1) | | 4 (22.2) | 1.000 |  |
| NPSLE | | 5 (11.4) | | 0 | | 5 (27.8) | 0.018 |  |
| Angiogenesis embolization | | 3 (6.82) | | 2 (7.69) | | 1 (5.56) | 1.000 |  |
| Lung | | 2 (4.55) | | 2 (7.69) | | 0 | 0.640 |  |
| SLEDAI-2K score |  | |  | |  | |  | |
| > 12 | 6 (13.6) | | 1(3.85) | | 5(27.8) | | 0.068 | |
| 4-12 | 34 (77.3) | | 21 (80.8) | | 13 (72.2) | | 0.765 | |
| 1-3 | 4 (9.09) | | 4 (15.4) | | 0 | | 0.226 | |

* Data are expressed as counted numbers (%). ^δ^Patients with probable PLE versus patients with definite PLE. GI: gastrointestinal; NPSLE: neuropsychiatric syndromes of SLE; SLEDAI-2K: Systemic Lupus Erythematosus Disease Activity Index 2000.

Supplemental table 2. Demographic characteristics and clinical data according to SLICC criteria in patients with definite and probable PLE*

|  | | Patients with probable PLE  (N=26) | Patients with definite PLE  (N=18) | | P |
| --- | --- | --- | --- | --- | --- |
| Female n (%) | 22(84.6) | | 15(83.3) | | 1.000 |
| Age, years (mean ± SE) | 32.7±2.86 | | 38.6±3.09 | | 0.179 |
| SLE Disease duration, months (mean ± SE) | 36.1±7.84 | | 50.7±20.7 | | 0.515 |
| PLE disease duration, months (mean ± SE) | 16.3±5.21 | | 5.36±1.14 | | 0.237 |
| SLICC criteria at admission n(%) |  | |  | |  |
| Acute cutaneous lupus | 3 (11.5) | | 3 (16.7) | | 0.626 |
| Chronic cutaneous lupus | 0 | | 1 (5.56) | | 0.852 |
| Oral ulcers | 0 | | 1 (5.56) | | 0.852 |
| Non-scarring alopecia | 2 (7.69) | | 3 (16.7) | | 0.661 |
| Synovitis | 3 (11.5) | | 3 (16.7) | | 0.968 |
| Serositis | 21 (80.8) | | 13 (72.2) | | 0.765 |
| Renal | 14 (53.8) | | 11 (61.1) | | 0.632 |
| Neurologic | 0 | | 5 (27.8) | | 0.018 |
| Hemolytic anemia | 1 (3.85) | | 0 | | 1.000 |
| Leukopenia | 3 (11.5) | | 3 (16.7) | | 0.626 |
| Thrombocytopenia | 4 (15.4) | | 3 (16.7) | | 1.000 |
| ANA | 26 (100) | | 18 (100) | | 1.000 |
| Anti-dsDNA | 8 (30.8) | | 2 (11.1) | | 0.244 |
| Anti-Sm | 2 (7.69) | | 0 | | 0.640 |
| Antiphospholipid | 0 | | 2 (11.1) | | 0.316 |
| Low complement | 26(100) | | 14 (77.8) | 0.047 | |
| Direct Coombs’ test | 3(11.5) | | 0 | 0.376 | |
| SLEDAI-2K score | 6.35±0.56 | | 10.3±1.51 | 0.024 | |

* Except where otherwise indicated, data are expressed as numbers (%). SLEDAI-2K: Systemic Lupus Erythematosus Disease Activity Index 2000; SLICC: Systemic Lupus International Collaborating Clinics.

Supplemental table 3. Laboratory findings in patients with definite and probable PLE*

|  | Patients with probable PLE  (N=26) | Patients with definite PLE  (N=18) | P | |
| --- | --- | --- | --- | --- |
| Platelet count (×10^9^/l) | 230.1±22.8 | 263.4±34.9 | 0.409 |  |
| Albumin, (g/l) | 16.1±0.96 | 17.9±1.35 | 0.255 |  |
| Plasma calcium (mmol/l) | 1.81±0.02 | 1.89±0.04 | 0.103 |  |
| Total cholesterol (mmol/l) | 7.28±0.66 | 6.87±0.61 | 0.665 |  |
| Triglyceride (mmol/l) | 4.31±0.95 | 2.51±0.27 | 0.129 |  |
| Serum C3 (g/l) | 0.46±0.04 | 0.52±0.07 | 0.352 |  |
| Serum C4 (g/l) | 0.10±0.01 | 0.10±0.02 | 0.742 |  |
| 24 hour urine protein (g/24 h) | 0.42±0.12 | 0.62±0.21 | 0.369 |  |
| Anti-SSA n (%) | 19(73.1) | 10(55.6) | 0.228 |  |
| Anti-SSB n (%) | 8(30.8) | 3(16.7) | 0.479 |  |
| Anti-RNP seropositive, n (%)¶ | 5(20.8) | 3(25.0) | 1.000 |  |

* Except where otherwise indicated, data are expressed as mean values ± SE. For anti-RNP, data were available for 24 patients with probable PLE, and 12 with definite PLE.

Supplemental table 4**.** Frequency of laboratory characteristics in the PLE and control groups.

| Group | PLE  (N=44) | Control  (N=88) | *P* |
| --- | --- | --- | --- |
| Platelet count > 300×10^9^/l | 12/41 (29.3) | 7/88 (7.95) | 0.001 |
| Albumin < 35 g/l | 44/44 (100) | 52/88 (59.1) | <0.001 |
| Plasma calcium < 2.13 mmol/l | 42/44 (95.5) | 39/88 (44.3) | <0.001 |
| Total cholesterol >5.7 mmol/l | 29/44 (65.9) | 36/88 (40.9) | 0.007 |
| Triglyceride > 1.7 mmol/l | 34/44 (77.3) | 54/88 (61.4) | 0.068 |
| Serum C3 < 0.6 g/l | 35/43 (81.4) | 47/84 (56.0) | 0.005 |
| Serum C4 < 0.12 g/l | 29/40 (72.5) | 46/81 (56.8) | 0.094 |
| 24-hour urine protein < 0.5 g/24 h | 35/44 (79.5) | 31/88 (35.2) | <0.001 |

Values are expressed as number n/N (%).

Supplemental table 5. Endoscopic and imaging findings from patients in the PLE group

|  | No. of patients n/N (%) |
| --- | --- |
| Endoscopic appearance | N=24 |
| Chronic superﬁcial gastritis and  generalized mucosal edema | 23/24 (95.8) |
| Normal looking | 1 |
| Computed tomography scan of abdomen | N=13 |
| Circumferential bowel wall thickening  with marked submucosal edema | 10/13 (76.9) |
| Abdominal cavity lymphadenopathy | 7 |
| Negative intestinal reconstruction | 3 |
| 99m Tc-labeled human serum albumin scan | N=18 |

Supplemental table 6. Treatment and prognosis of PLE patients

|  | PLE patients  n(%) |
| --- | --- |
| Treatment | N=44 |
| Corticosteroids | 44 (100) |
| MP pulse therapy | 14 (31.8) |
| Immunosuppressive agents | 40 (90.9) |
| CTX | 37 |
| MMF | 1 |
| CTX+MMF | 1 |
| MTX | 1 |
| Treatment response | N=18 |
| 2 months |  |
| CR | 8 (44.4) |
| PR | 4 (22.2) |
| NR | 6 (33.3) |
| 3 months |  |
| CR | 13 (72.2) |
| PR | 4 (22.2) |
| NR | 1 (5.56) |
| 6 months |  |
| CR | 16 (88.9) |
| PR | 1 (5.56) |
| NR | 1 (5.56) |

CR: complete response CTX: Cyclophosphamide; MMF: mycophenolate mofetil; MP: methylprednisolone; MP pulse therapy: 1g MP for 3 days and then shifted to 1mg/kg/d; MTX: methotrexate; NR: non-response; PR: partial response.
